# Grazed wet meadows are sink habitats for the southern dunlin (*Calidris alpina schinzii*) due to nest trampling by cattle

**DOI:** 10.1002/ece3.2369

**Published:** 2016-09-09

**Authors:** Veli‐Matti Pakanen, Sami Aikio, Aappo Luukkonen, Kari Koivula

**Affiliations:** ^1^ Department of Ecology University of Oulu PO Box 3000 FIN‐90014 Oulu Finland; ^2^ Finnish Museum of Natural History Botany Unit University of Helsinki PO Box 7 FIN‐00014 Helsinki Finland

**Keywords:** conservation, dispersal, management, nest trampling, recruitment, restoration, shorebird

## Abstract

The effect of habitat management is commonly evaluated by measuring population growth, which does not distinguish changes in reproductive success from changes in survival or the effects of immigration or emigration. Management has rarely been evaluated considering complete life cycle of the target organisms, including also possible negative impacts from management. We evaluated the effectiveness of cattle grazing in the restoration of coastal meadows as a breeding habitat for small and medium‐sized ground‐nesting birds by examining the size and demography of a southern dunlin (*Calidris alpina schinzii*) breeding population. Using a stochastic renesting model that includes within‐season variation in breeding parameters, we evaluated the effect of grazing time and stocking rates on reproduction. The census data indicated that the population was stable when nest trampling was prevented, but detailed demographic models showed that the population on managed meadows was a sink that persisted by attracting immigrants. Even small reductions in reproductive success caused by trampling were detrimental to long‐term viability. We suggest that the best management strategy is to postpone grazing to after the 19th of June, which is about three weeks later than what is optimal from the farmer's point of view. The differing results from the two evaluation approaches warn against planning and evaluating management only based on census population size and highlight the need to consider target‐specific life history characteristics and demography. Even though grazing management is crucial for creating and maintaining suitable habitats, we found that it was insufficient in maintaining a viable population without additional measures that increase nest success. In the presently studied case and in populations with similar breeding cycles, impacts from nest trampling can be avoided by starting grazing when about 70% of the breeding season has past.

## Introduction

European Union (EU) agri‐environment schemes (AES) attempt to halt long‐term declines in farmland biodiversity by investing in substantial incentives paid for farmers to change farming practices (Kleijn et al. 2006, 2011; Wilson et al. 2007). These actions are important for the conservation of grassland flora and fauna but sometimes goals are not achieved (Kleijn et al. 2006, 2011; Wilson et al. 2007; Kentie et al. 2013, 2015; Smart et al. 2014). One scheme includes the re‐establishment of livestock grazing on open coastal meadows that have deteriorated or disappeared following the cessation of traditional agricultural practices due to economic reasons (Ottvall and Smith 2006). These large‐scale actions are particularly important for birds and especially for waders, which constitute a large portion of avifauna on such grasslands. In some cases, grazing management has successfully increased suitable breeding habitat with a concurrent increase in breeding densities of many species (e.g., Olsen and Schmidt 2004; Ottvall and Smith 2006; Żmihorski et al. 2016).

However, evaluations of the effectiveness of the AES based on correlating management intensity with species richness or density are susceptible to bias from unconsidered ecological phenomena (e.g., Filippi‐Codaccioni et al. 2010; Kleijn et al. 2011). When evaluating the general response of bird diversity to grazing in farmlands and grasslands, these approaches may be misleading because species such as waders may prefer pastures that provide more attractive sward structure than ungrazed meadows (Durant et al. 2008). Thus, the evaluations of AES may be biased due to underlying bird movement and source–sink dynamics, and the observed responses in, for example, population density may therefore reflect an influx of immigrants from other sites rather than improved management result (Pulliam 1988; Brawn and Robinson 1996). Successful evaluation of management should therefore distinguish whether increased density reflects improved local reproductive success or increased immigration (Kleijn et al. 2011; Pakanen et al. 2011).

Sometimes management may also have negative effects. For example, grazing may reduce local recruitment through associated disturbances, for example, nest trampling (Beintema and Müskens 1987; Hart et al. 2002; Mandema et al. 2013; Sharps et al. 2015; Sabatier et al. 2016). Trampling rates can be high and may even threaten the viability of populations (Watson et al. 2006; Pakanen et al. 2011). In such cases, pastures may be sink habitats for birds where local recruitment is insufficient in maintaining the populations (Pulliam 1988; Donovan et al. 1995). Because grazing management is vital for maintaining biodiversity and is becoming an increasingly popular management option for grasslands, it is important to evaluate its positive and negative impacts, and to find out the best solutions for maintaining biodiversity (Sabatier et al. 2015).

Despite the existence of data on the effects of livestock on reproductive success of waders (see above studies), the evaluation of its consequences to population viability is difficult if other demographic parameters are ignored. A demographic approach with population modeling enables both the evaluation of management and the identification of optimal grazing practices (Sabatier et al. 2010; Kleijn et al. 2011). However, such studies require complete and population‐specific life history data, and are therefore rare (but see Rolek et al. 2016). So far, no study has considered within‐season temporal factors in breeding parameters (phenology, replacement nesting, nest survival, and juvenile survival) when analyzing livestock effects on population viability. Considering within‐ and between‐season variation in these parameters is especially important, because overlap in the timing of grazing and breeding influences trampling rates, and because trampling rates are highest at the beginning of the grazing season (Durant et al. 2008; Pakanen et al. 2011).

**Figure 1:**
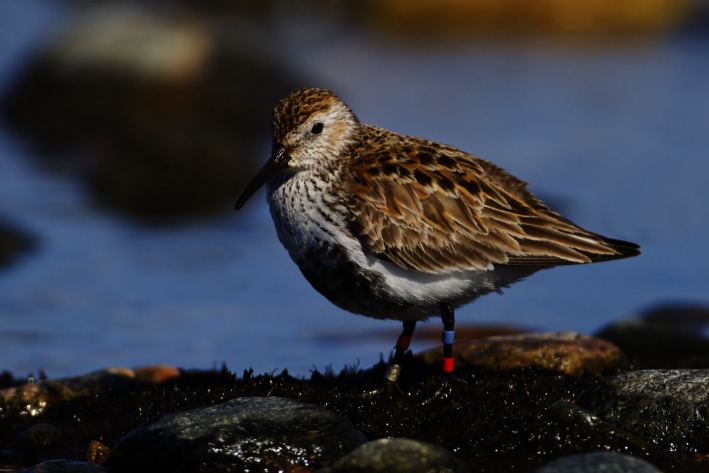
Color‐ringed male southern dunlin (*Calidris alpina schinzii*) photographed during spring migration in Jurmo, Finland. © Jorma Tenovuo.

In this study, we evaluate the effects of grazing as a management tool for small and medium‐sized ground‐nesting birds using the southern dunlin (*Calidris alpina schinzii*, hereafter dunlin) as a model species (Fig. 1). Because of its preference to short sward (Thorup 1998), the critically endangered Baltic population of the dunlin breeds almost exclusively on low and wet coastal pastures that have distinctively shorter grass than unmanaged meadows. The Baltic metapopulation of the dunlin has suffered in recent decades due to degradation of habitats, nest predation, inbreeding, and consequent low reproductive success (Jönsson 1991; Thorup 1998, 2006; Blomqvist et al. 2010). Consequently, the metapopulation has declined from about 2500 pairs to only about 500 pairs in few decades (Thorup 2006; Helcom 2013). In contrast to all other subpopulations, the decline of the Finnish dunlin population has recently halted, which is considered to be an example of successful grazing management (Rassi et al. 2010). We evaluate the effectiveness of grazing using both census‐ and demography‐based approaches. In the latter approach, we use long‐term individual‐based data encompassing complete life history (i.e., local recruitment, survival, and immigration) to (1) disentangle the life history traits behind the observed population growth and to (2) evaluate whether demography of this population depicts that of a sink or a source (Pulliam 1988). We then (3) quantify reproductive success under different regimes of grazing time and stocking rates using a stochastic renesting model that accounts for within‐season variation (e.g., Beintema and Müskens 1987) in parameters that affect local recruitment (phenology, renesting, nest survival, nest trampling, hatching success, and juvenile survival). Finally, we model the effects of nest trampling on population viability under varying grazing practices to draw guidelines for population management.

## Methods

### Study area and population

**Figure 2:**
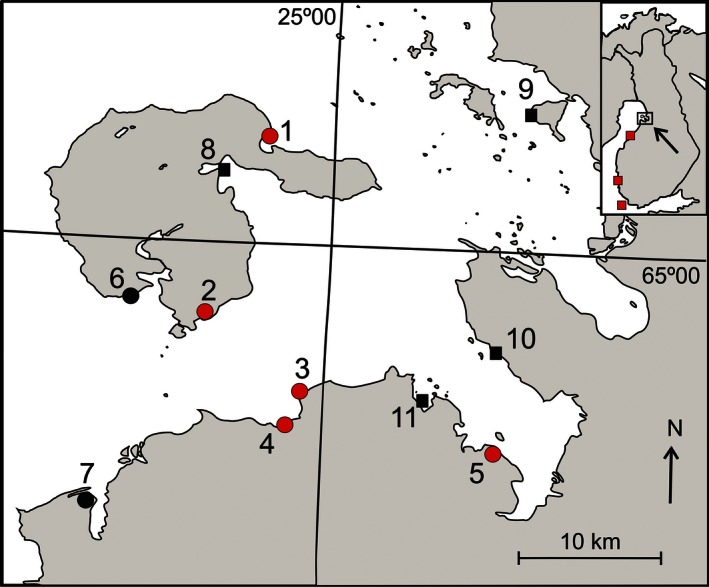
The location of the study population at Bothnian Bay, Finland, and other known breeding sites. The breeding sites under intensive study are marked with numbered round symbols. Sites 1–5 (red) were grazed, and sites 6–7 (black) were mowed during the study. Sites 8–11 (black squares) were only censused annually for breeding dunlin. These sites consisted between 0 and 1 pairs. Other breeding sites (red squares from north to south: Kalajoki, Pori, and Jurmo) are indicated in the map of Finland. Modified from Pakanen (2011).

Our study system is the northernmost subpopulation of the critically endangered Baltic dunlin metapopulation, which still exists on the coasts of Denmark, Sweden, Germany, Estonia, and Finland (Helcom 2013). Our study area that has about 45 pairs is situated on the coast of the northern Baltic Sea, the Bothnian Bay (*c*. 64° 50′ N, 25° 00′ E), and it is separated from the nearest breeding sites by hundreds of kilometers (Fig. 2). Other breeding areas in Finland are in Kalajoki (0–2 pairs during the study), Pori (4–5 pairs), and Jurmo (4–5 pairs; Fig. 2). The closest larger populations to Finland are in Estonia. The alpina subspecies migrate through the Bothnian Bay, and the closest populations breed about 400 km north from our study area in Enontekiö. The subspecies can be distinguished from each other from plumage characteristics, and we have not observed mixed pairs of these (own observations).

We studied breeding dunlin on seven breeding sites. These sites varied in size from 27 ha to over 500 ha and pair numbers of dunlin varied from 1 to 29 (Fig. 2). The vicinity includes four occasionally inhabited breeding sites (0–2 pairs; Fig. 2), which were regularly checked for breeding dunlins. In this study, we used data collected from five pastures (Fig. 2). The remaining two sites were managed by mowing and were excluded from this study to concentrate on the effects of grazing. Pastures were grazed with large beef cattle breeds such as the Limousine. Grazing pressures varied between years and sites but were between 0.5–1 livestock units/ha as recommended for suckler cows on coastal meadows. Grazing was commenced variably between pastures with the earliest starting in late May. Livestock were usually introduced to the pasture during one day, and they were kept there until autumn. The pastures included only one large area that was surrounded by a fence or the shoreline. Since the start of the study (2002), nests have been protected against trampling with steel arches that do not protect against predators (Pakanen et al. 2011). This provides a basis of life history parameters under management to which effects of trampling will be added with the renesting model (see below).

### Life history data

Life history data were collected from 2002 to 2010. Territories and nests were searched from late‐April to mid‐July. Nests with eggs were considered active and were visited every one to seven days until the nest fate was known. Nest ages and timing of breeding were determined by the egg number during laying, egg floatation, hatching date, or size of chicks (Pakanen et al. 2011). Birds were considered to be renesting when they had laid a replacement nest after losing a nest (Pakanen et al. 2014).

Adult birds were captured with walk‐in traps or mist nets when incubating or brooding hatchlings. Adults were given individually identifiable color ring combinations on their tarsi (metal ring and three darvic color rings). Hatchlings were ringed with a metal ring only. Resightings of color‐ringed birds were considered recaptures. Individuals were sexed according to the CHD gene (Griffiths et al. 1998), sexual behavior, or morphological measurements. Fieldwork was carried out with permission from the North Ostrobothnian regional environment center (PPO‐2004‐L‐289‐254, PPO‐2006‐L‐206‐254) and complied with national laws.

### Analysis of nest survival and capture–recapture data

We modeled daily nest survival (Dinsmore et al. 2002), apparent survival (Φ; survival estimate corrected for recapture probabilities) of adults and juveniles with the Cormack–Jolly–Seber model (CJS; Lebreton et al. 1992) in program MARK (White and Burnham 1999). We used AIC model selection in nest survival and apparent survival analyses following Burnham and Anderson (2002). Survival estimates were derived by averaging the results from models ΔAIC ≤4 to account for the uncertainty in model selection. See Appendix S1 for detailed descriptions of the modeling approaches.

### The renesting model

Dunlin may lay replacement nests after a failure earlier in the season (Pakanen et al. 2014), which we considered when calculating the proportion of successful breeding attempts, number of hatchlings produced, and local recruitment per female with a stochastic simulation model (renesting model, see Beintema and Müskens 1987; Ratcliffe et al. 2005). The model is described in detail in Appendix S2 and was constructed in MATLAB (The MathWorks Inc., Natick, MA). We modeled all possible parameters in the breeding cycle that affect reproductive success from start of egg laying to recruitment for individual females (replicates) while considering date and its effect on each parameter. The replicates were then used to calculate averages.

We used the renesting model for calculating reproductive performance for management scenarios of varying trampling rates and grazing initiation dates. Scenario 1 assumed the absence of trampling. For scenarios including trampling, we used trampling probabilities calculated for species exhibiting no active defense against cattle (Beintema and Müskens 1987; Thorup 1998; Pakanen et al. 2011). Scenario 2 represents low stocking rates of 0.5 head/ha, which corresponds to a daily trampling rate of 0.02 (i.e., a 2% daily probability for a given nest to be trampled). Scenario 3 represented moderate stocking rates of 1 head/ha, which we estimate to cause a daily trampling rate of 0.04. Finally, scenario 4 included high stocking rates with an average trampling rate of 0.067, which is caused by stocking rates of 1.72 head/ha (see fig. 3 in Pakanen et al. 2011). See Appendix S2 for more information on modeling trampling rates.

We present the results along calendar dates and percent advancement of the breeding season, which is considered to be the period from the first to the last observed nest. The phase of the season refers to the percentage of the breeding season that has past (not to be confused with the percentage of nests active).

### Estimation of population growth rates

We estimated population growth rates from capture–recapture data using (1) pair count data (λ_CENSUS_ = *N*_t+1_/*N*_t_), 2) temporal symmetry models (λ_PRADEL_, Pradel 1996), and (3) a stage‐structured projection matrix model (λ_MATRIX_, Caswell 2001). We estimated the average λ_CENSUS_ as the geometric mean of growth rates between subsequent years in the census data.

We used temporal symmetry models to estimate λ_PRADEL_ and the recruitment parameter (f; Pradel 1996) in MARK. The recruitment parameter gives the number of individuals entering the population at time *i* + 1 per adult individual already in the population at time *i* (Nichols et al. 2000). Both sexes were included in the analyses. We examined goodness of fit for the fully time‐dependent global model, Φ(t)p(t)λ/f(t), based on the test for the CJS model (GOF_BOOTSTRAP_, *P* = 0.256, ĉ = 1.08, Sandercock and Beissinger 2002). We calculated λ_MATRIX_ as the dominant eigenvalue of a modified Lefkovich matrix model that was based on a prebreeding census and described female dynamics with stages representing three age groups with the last group containing ages three or older (see Appendix S3).

We evaluated whether the dunlin population on the grazed meadows was stable, a sink, or a source (Pulliam 1988) by comparing population growth rate estimates λ_PRADEL_ and λ_CENSUS_, which both include complete demography, to λ_MATRIX_, which does not include immigration (Peery et al. 2006). Thus, if λ_PRADEL_ and λ_CENSUS_ ≥1 and the λ_MATRIX_ <1, the population is likely to be a sink kept alive by immigration (Peery et al. 2006). In case we found this pattern, we evaluated the possibility that the result is caused by emigration, that is, a pseudo sink. This was carried out by examining the potential combinations of juvenile and adult emigration rates that would indicate a stable population or a net exporting population, that is, a source (Runge et al. 2006), and compare these to what is known about dispersal propensities of dunlin. We present emigration rates in relation to all individuals alive at time t, which makes emigration comparable with survival, for example, a 0.1 emigration rate and mortality rate both amount to the same reduction in the population growth rate.

### Assessing management with extinction risks

We used RAMASmetapop (Akçakaya 2005) to evaluate extinction risks in the next 20 years under grazing scenarios 1–4 using the matrix model described above (See Appendix S3). We used a threshold of 30% decline to examine population viability when immigration was included in the model. When immigration was not included, we used an extinction threshold of one individual.

## Results

### Reproduction

The start of egg laying peaked in mid‐May (median: May 13th, mean 14.2 ± 7.11 [SD] days since May 1st), while laying of replacement clutches started in late May (median: June 1st, mean: 31.55 ± 6.42 [SD] days since May 1st; Appendix S4, Fig. S3). Renesting probability decreased with the advancement of the breeding season (Appendix S4, Fig. S4). The re‐laying interval between nest failure and the first egg of the replacement clutch was 4.85 ± 1.75 (mean ± SD, *n* = 24) days. Clutch size did not differ between first (3.85 ± 0.48, *n* = 260) and replacement nests (3.68 ± 0.55, *n* = 28), neither did the number of hatched chicks per successful nest (3.57 ± 0.74, *n* = 155 vs. 3.53 ± 0.74, *n* = 15).

### Daily nest survival

Daily nest survival (277 nests, 2932 nest days) varied between years (Table S1, models A3 vs. a10, ΔAIC = 22) ranging from 0.897 to 0.987 (Fig. S5), which translates to a nest success from 6% to 71%. There was a strong interaction between nest age and year (Table S1, models A1 vs. A4, ΔAIC = 17; Appendix S4). Nest survival increased with nest age in most years but decreased in 3 years (Appendix S4, Fig. S6). Nest type (first nest vs. renest) did not affect nest survival when year and age effects were controlled (Table S1). Mean daily nest survival (from all causes except trampling) was 0.971 ± 0.003, which results in a 46.5% probability to survive over the 26‐day nesting period.

### Juvenile survival and age of first breeding

Juvenile survival from hatching to their first summer was on average 0.20 (±0.034; Fig. S7). Juvenile survival decreased strongly with the advancing hatching date during the breeding season (Table S1, models B1 vs. B2, ΔAIC = 3.72, β_HATCH_ −0.052, 95% CI [−0.096, −0.008]; Fig. S8). Adult survival of local recruits was high (0.89 ± 0.053). Recapture probabilities were age specific (Table S1, ΔAIC >80, Model B13 vs. models B2–B3, B6). A three‐age‐group structure (1‐year‐, 2‐year‐old, and a pooled group for 3 years and older) was most supported. Model averaged recapture rates increased with age until the third year (ac1, 0.013 ± 0.0134; ac2, 0.480 ± 0.079; ac3, 0.768 ± 0.062). Corresponding breeding probabilities (=ac*i*/ac3) for different ages were 1.7%, 62.5%, and 100%, respectively.

### Apparent adult survival

Apparent adult survival rates were constant in time (Table S1, ΔAIC = 9.7), being 0.77 (±0.022) on average (see Fig. S9 for annual estimates). Sex and time since marking were included within the best models, but their effects were weak being *c*. 1.17 ΔAIC units from the constant model. Therefore, model averaged survival estimates did not vary much (first‐time breeders: males 0.76 [±0.032], females 0.75 [±0.034], experienced breeders: males 0.78 [±0.026], females 0.76 [±0.029]). Recapture probabilities were high, constant in time, and similar between males (0.89 ± 0.23) and females (0.88 ± 0.027).

### Renesting model

**Figure 3:**
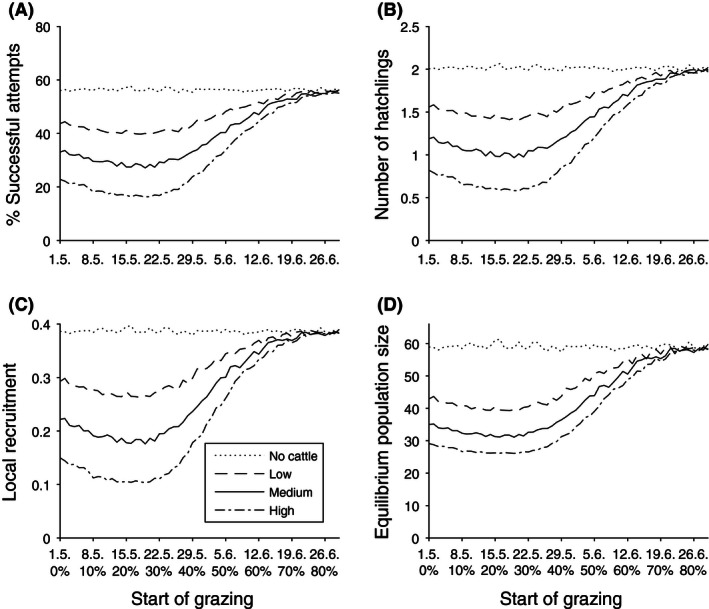
(A) The percentage of failed nesting attempts, (B) number of hatchlings, (C) local recruitment per female, and (D) predicted equilibrium population sizes (females) under different intensities and starting dates of grazing. Start of grazing (*x*‐axis) is represented by date (May 1st = 1) and the percentual advancement of the breeding season.

When trampling was excluded, 56.3% of females were successful in hatching a nest during the breeding season (Fig. 3A). Each female produced on average 2.01 hatchlings and 0.387 one‐year‐old offspring per breeding attempt (Fig. 3B and C). The effect of trampling on reproductive success was dependent on the stocking rates and timing of grazing (Fig. 3). If grazing started before the mid‐breeding season (peak incubation stage, latter half of May) with high stocking rates (1.72 head/ha), local recruitment declined 73% when compared to conditions with no cattle (Fig. 3C). Even with trampling rates corresponding to low or moderate stocking rates (0.5 or 1 head/ha), local recruitment decreased 53% or 31%, respectively. During the latter half of May, most of the first nests are active (Appendix S4, Fig. S3). The effect of trampling on reproductive success gradually decreased during the mid‐breeding season in late May to early June, when 40–60% of the breeding season has passed. If the introduction of cattle to the pasture was postponed to the time after the hatching peak (about 70% of the season), when only *c*. 25% of all nests are active, effects on reproductive success were markedly reduced (Fig. 3).

### Population growth rates and immigration

Due to insufficient catching of individuals prior to 2005, estimates from Pradel models were calculated from 2005 to 2010. In order to be able to retrieve annual estimates, we kept the population growth rate and recruitment time‐dependent. The best model included constant survival and recapture rates. Population growth calculated with capture–recapture models (λ_PRADEL_ = 1.019 ± 0.027, CI 0.949–1.089) and from pair numbers (λ_CENSUS_ = 1.00 ± 0.089, CI 0.897–1.013) both suggest a rather stable or a growing population. In contrast, the population growth rate calculated from the matrix model (λ_MATRIX_ = 0.912 ± 0.036, CI 0.829–0.993) was clearly less than one and suggested that in the absence of immigration, the population would annually decline by 8.8%. Emigration rates needed for the population to be a source were high (adults 0.1 and juveniles 0.15) when compared to published natal and breeding dispersal studies (Soikkeli 1970a; Jackson 1994; Thorup 1999; Flodin and Blomqvist 2012), suggesting that the population is likely to be a sink (Appendix S4, Fig. S10). The stability of the population with the observed adult survival (0.76) and juvenile survival (0.2) would require nest success to be around 82%.

Total recruitment (f, recruitment parameter) was sufficient to maintain stable growth, as on average, 0.259 (±0.029) individuals recruited each year for every individual in the population in the previous year. However, the proportion of immigrants among the recruits was large. Based on field observations, the number of immigrants each year (9.75 ± 1.65, consisting 4.00 ± 0.56 males and 5.75 ± 1.25 females) was similar to the number of local recruits (born between 2002 and 2008) each year (8.75 ± 1.65, consisting of 3.75 ± 0.85 males and 5.00, ±1.22 females). The number of immigrants during the whole study included eight juveniles that had dispersed from the two nongrazed (mowed) meadows to grazed areas. Movement of adults was rare. On average, one adult individual (±0.463, *n* = 8 years) dispersed between breeding sites per year, including four adults that dispersed from the two nongrazed meadows to grazed areas. Thus, 12 immigrants were known to have originated from the local breeding population in the Bothnian Bay.

### Population viability and trampling

**Figure 4:**
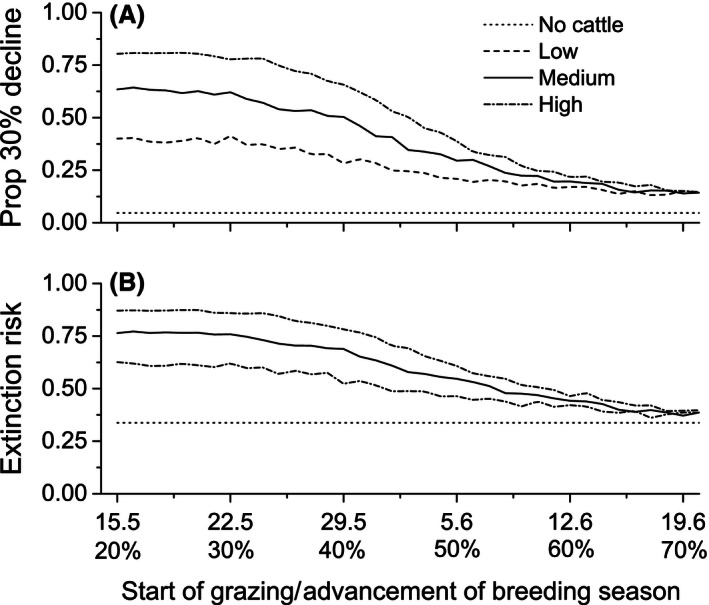
Predicted risks of population decline in the next 20 years in relation to different grazing scenarios (no trampling, low, moderate, and high trampling rates) and the timing of grazing. (A) Probability of 30% decline when immigration is considered and (B) extinction risks with no immigration.

Projections including constant immigration had a 4.6% (95% CI 3.7–5.5) probability of a 30% decline within the next 20 years (Fig. 4A), and the equilibrium population size was estimated at 60 females (Fig. 3D). Extinction risk (one individual threshold) was relatively high 33.7% (95% CI 32.8–34.6) when immigration was excluded (Fig. 4B).

Trampling of nests resulted in a declining population if grazing started early (Figs 3D and 4). Without immigration, all grazing scenarios led to high extinction risks within the next 20 years (Fig. 4B). When immigration was considered, the effect of trampling on population viability remained small, and was most pronounced if grazing started in May, before the mid‐breeding season (Fig. 4A). The effect of trampling was most severe under high stocking rates, where the probability of a 30% decline peaked at 80%. Under high stocking rates, the probability of 30% decline decreases to 15% if grazing is postponed to start after the mid‐breeding season (June 19th, Fig. 4A).

## Discussion

Grazing management is crucial for creating and maintaining suitable habitat for small and medium‐sized ground‐nesting birds, but our life history data and models show that it may not provide high enough breeding success to maintain viable populations in the long run. Census population size and capture–recapture models indicated stable population growth for the dunlin population under grazing management where nests were protected from trampling. At the first glance, this would appear as a successful case of dunlin conservation, but demographic models with survival and fecundity, but excluding immigration, indicated an annual population decline of *c*. 9%. The difference between these population growth estimates reveals our study population as a sink, dependent on immigrants entering the managed meadows (Pulliam 1988; Dias 1996; Peery et al. 2006). Our projections thus predict an imminent danger of extinction unless rescued by immigration. The different results between these approaches illustrate the importance of considering full life history when planning and evaluating the effectiveness of management.

Our models show that trampling is detrimental to long‐term viability of the dunlin population even under low grazing pressures, especially if grazing started too early in the breeding season. Thus, measures are needed in order to reduce these negative impacts. Nest protection is effective in reducing trampling as shown in our study (Pakanen et al. 2011). However, nest protection requires finding all the nests which may be unfeasible in most cases. The other option is to adjust the timing and intensity of grazing. Here, managers fall into trade‐off situations where improvement in an aspect of a target organism's environment or life cycle might be associated with deterioration of other aspects (Sabatier et al. 2010). Trampling can be avoided primarily by reducing stocking rates or by postponing grazing to a later phase of the growing season. Finding the optimal grazing pressure is not only an issue of trampling. It is further complicated by different management goals, for example, maintaining suitable sward height and the farmer's interests (Tichit et al. 2007; Durant et al. 2008; Sabatier et al. 2010). A reduction in stocking rates results in lowered trampling rates, but as shown, even the use of low stocking rates (e.g., 0.5 head/ha) during mid‐incubation season can still result in a large reduction in hatching success. Furthermore, low stocking rates may not be capable of maintaining the sward sufficiently low, which may in turn lead to a reduction in habitat attractiveness and possibly decreased breeding success for small ground‐nesting birds.

An overlap between grazing and breeding phenology, and temporal patterns in trampling, nest success, renesting probability, and local recruitment, emphasized the impact of nest trampling in early season, making the start of grazing one of the most crucial aspects of management. In case no information exists on complete demography of a target species, as the situation usually is, the safest strategy would be to start grazing as late as possible. We found the risks of population decline to be highest when grazing was started before the mid‐breeding season. After that, the proportion of active nests gradually decreases reducing the effect of livestock. If grazing is started after 70–80% of the breeding season has passed (*c*. 19th – 26th of June at Bothnian Bay) when on average <25% of nests (mostly replacements) are active, trampling no longer significantly harms reproductive success. Because there is considerable variation in timing of breeding due to variation in weather, the date of a safe start to grazing may differ. However, because dunlin significantly reduce egg laying in June and stop by mid‐June (Fig. S3), and because juvenile survival crashes with the season (Fig. S8), the effects of trampling are small even in late years if grazing is started after the 26th of June.

A later start with emphasis on late summer grazing would enable the safe use of higher stocking rates. This would produce lower and thus more suitable sward height for settling birds in the early spring (Tichit et al. 2005; Durant et al. 2008). Postponing grazing may, however, also have detrimental effects on the vegetation and thus habitat suitability. In case late onset of grazing results in a sward that is too tall for attracting small ground‐nesting birds, additional measures such as mowing are warranted late in the season.

A late start to grazing may not satisfy cattle feeding requirements and may increase costs (Sabatier et al. 2010). Whereas spring grazing before the breeding season can be a solution in temperate regions, for example, central Europe (Durant et al. 2008), it is difficult in northern regions due to required extra feeding. The most suitable time for initiating grazing for beef production at our study area would be late May (i.e., mid‐breeding season for dunlin and most other meadow birds), which means that there is a strong conflict of interest between conservation goals and the benefits of cattle owners. Grazing should not start later than mid‐June to ensure high‐forage quality (Niemelä et al. 2008). This conflict could be avoided by the early grazing only taking place in those areas that are least suitable for the managed species.

Our results and recommendations on grazing management can be extended from dunlin to several other bird species breeding on pastures and having similar timing and habitat requirements. At Bothnian Bay, dunlin breed more or less at the same time as lapwings (*Vanellus vanellus*), black‐tailed godwits (*Limosa limosa*), and redshanks (*Tringa totanus*), but earlier than most ruffs (*Calidris pugnax*) and especially Temminck's stints (*Calidris temminckii*) and arctic terns (*Sterna paradisaea*), which have their hatching peaks in late June (own observations). Optimal time for grazing is in July for these late breeding species. A generally low, but variable sward height would provide suitable habitat for most of these species (Durant et al. 2008).

### A sink population?

We found that local recruitment was insufficient for compensating the loss of adults in mortality or emigration, suggesting a sink population status (Pulliam 1988). Low recruitment can be attributed partly to poor nest success even when losses to trampling were not included, but we cannot exclude the possibility that, for example, adult survival or juvenile survival have declined (e.g., Pakanen and Thorup 2016). Excluding our study population, the Baltic dunlin populations declined at an 8% annual rate during 2000–2010 based on data from Helcom (2013), which is in agreement with matrix model projection for our study population. However, in contrast to the declining southern populations, our study population appeared stable because immigration compensated for the loss of adults.

What is the source of immigration and what is the nature of these populations in terms of source–sink theory? Some immigrants can be locally born and originate from few unnoticed breeders (see Methods). However, most individuals must be true immigrants because low reproductive success of dunlins means that the number of pairs needed to produce annually eight recruits is so large (*c*. 25 pairs) that it cannot remain unnoticed in surveys. If half of the recruits return to their natal site, *c*. 50 pairs would be needed to produce the observed annual immigration, and the requirement would be much higher if the origin of immigrants is hundreds of kilometers away in the southern parts of the breeding range. Given the rapid population decline of the Baltic dunlin, the most probable sources of immigrants to our study populations are likely to be sinks themselves, where poor environmental conditions lead to increased rate of dispersal.

In a sink population, the per capita rates of emigration and immigration are unbalanced (Diffendorfer 1998), and sampling therefore has to cover the whole metapopulation in order to ascertain correct interpretation of source–sink dynamics (Runge et al. 2006). Because immigrants exist in our study population, emigration is also a possible explanation for declining population sizes. While we cannot exclude emigration due to dispersal being a built in trait especially among juveniles, several facts suggest that permanent emigration has a minor effect on our survival estimates. Thus, our population may be a so called leaky sink, where some emigration may exist while immigration is a more dominant factor (Dias 1996).

Firstly, molecular data indicate an existing gene flow between populations in Bothnian Bay and southern Baltic, such that movement from the southern Baltic to Bothnian Bay is stronger than from Bothnian Bay to the south (Rönkä N. et al., unpubl. data). Indeed, our study population has received ringed individuals hatched in Pori 400 km away, but none originating from Bothnian Bay have been seen outside their native area despite regular censuses and trapping.

Secondly, our study species is constrained to short vegetated meadows that exist only in actively managed sites (mowing or grazing). This allowed us to control for small‐scale movement, as our census covered virtually all suitable habitat within our geographically large study area that is isolated from the other breeding sites in the southern parts of the Baltic Sea (Fig. 2). Our study area extends well over the dispersal distributions described for dunlin (adults: range 0–5 km, juveniles range: 0.07–16 km, Soikkeli 1970a; Jackson 1994; Thorup 1999; Flodin and Blomqvist 2012). For example, half of the returning juveniles (28 recruits) dispersed between study sites with an average dispersal distance of 18.5 km suggesting a good coverage for dispersal (Pakanen V.‐M. et al. unpubl. data). This advantage of the current study contrasts with many other studies, which are characterized by small study areas among large breeding ranges (Zimmerman et al. 2007).

Thirdly, we evaluated total emigration rates needed for the population to be a source (net exporter) for different combinations of juvenile and adult survival (Fig. S10 in Appendix S4). Our high estimate of juvenile survival suggests that fidelity to the natal region (within 40 km) is strong. Apparent juvenile survival (0.20) is considerably higher than reported for many other small wader species (range 0.048–0.1, Koivula et al. 2008; Nol et al. 2010; Pakanen et al. 2015) but close to true survival (0.179, corrected for permanent emigration) estimated for snowy plovers (*Charadrius alexandrinus nivosus*, Stenzel et al. 2007), indicating that our estimate is realistic. Hence, the increase in survival to 0.35 that is needed for the population to be stable (Appendix S4, Fig. S10) is unrealistic. Indeed, survival after fledging was estimated to be 0.50 in a Swedish dunlin population indicating that most mortality occurs before fledging (Jönsson 1991).

Adult dunlins are very site faithful, and consequently, our estimates of apparent adult survival should be reliable. Our estimate (0.76) is comparable to other dunlin studies (return rates: Jönsson 1991, 0.83; Soikkeli 1970b, 0.741; apparent survival: Ryan et al. 2016, 0.72; Pakanen and Thorup 2016, 0.79). None of these estimates would be sufficient for stable population growth with the observed reproductive success. Adult survival should be >0.85 for the population to be stable. Because even movements between the study sites inside Bothnian Bay are rare (1 movement/year), such strong permanent emigration (8–9 individuals yearly) outside the study area is unlikely.

Finally, let us consider a scenario where the population would be stable. This could be achieved if adult emigration would be 0.1 or juvenile emigration would be 0.15 (Fig. S10). This would mean that adult survival would actually be 0.86 or juvenile survival would be 0.35. Annually, either of these would result in about four to five females emigrating from our study area (and possibly males too). Yet, none of our ringed birds have ever been seen breeding outside Bothnian Bay even though these populations are followed intensively, some even as closely as our study population.

What could explain the unbalanced movement of individuals between dunlin populations? These populations may not have different levels of site fidelity, but rather some ecological factors may explain the observed difference. It is possible that the long‐distance dispersal of juveniles to the north is facilitated by the phenological difference; birds arriving from the south (that follow the southern phenology in their timing of migration) have time to disperse to the north but those juveniles that originate from the north (that follow the northern phenology in their timing of migration) are late for breeding when they fly over the southern breeding grounds (Pakanen V.‐M. et al. submitted). Furthermore, the southern populations are many times larger than our study population and the number of long‐distance dispersers is therefore larger than in our study population.

## Conclusions

Grazing restored and maintained habitat for breeding dunlin and without a doubt improved their reproduction. While the grazed habitats generally appeared to be sinks, pastures may well be sources during years of good nesting success (Johnson 2004) and when trampling is prevented by late grazing. Therefore, other measures such as predator control may be required for keeping populations stable without relying on immigration. Importantly, our models revealed that an early timing of grazing with high stocking rates further decreased viability through nest trampling. Based on their relatively high immigration rates, managed pastures may be more attractive than other meadows, possibly turning these sink habitats into ecological traps (Kristan 2003; Battin 2004). Our results, therefore, underline the need for specific consideration of life history of the managed species when planning and evaluating management and the need to time grazing so that it does not coincide with the breeding time of birds. The economical and habitat quality consequences of postponed grazing need to be considered.

## Data Accessibility

Pair numbers, capture–recapture data, and nest data are available in DRYAD: DRYAD entry doi:10.5061/dryad.b2852.

## Conflict of Interest

The authors declare no conflict of interest.

## Supporting information


**Table S1.** Result from capture–recapture and nest survival modeling.

**Appendix S1.** Methods on capture–recapture and nest survival models.

**Appendix S2.** Description of the renesting model.

**Appendix S3.** Description of the matrix model and the PVA.

**Appendix S4.** Results as figures.
