# Supplementary material for: Grazed wet meadows are sink habitats for the southern dunlin (Calidris alpina schinzii) due to nest trampling by cattle
Source: Ecol Evol. 2016 Sep 9;6(20):7176–87. doi: 10.1002/ece3.2369 (PMC5513266; doi:10.1002/ece3.2369)
Supplement: Supplementary file 1 — Table S1. Result from capture–recapture and nest survival modeling. [file ECE3-6-7176-s001.docx]

Supplementary Material, Table S1

Pakanen, V.-M., Aikio, S., Luukkonen, A. & Koivula, K. (2016) Grazed wet meadows are sink habitats for the southern dunlin (*Calidris alpina schinzii*) due to nest trampling by cattle. *Ecology and Evolution*

Table S1. Results of models for analyzing A) nest survival, B) juvenile survival (Φ), C) adult survival (Φ), D) population growth rate (λ) and the recruitment parameter (f) of the Southern dunlin (*Calidris alpina schinzii*) breeding in Finland. For each model we report model number (No., refers to the numbers used in the text), the model structure (see footnote), Akaike’s information criterion corrected for small sample size ([Q]AICc), difference in (Q)AICc compared to the best fitting model (QAICc), Akaike weights (w) and number of parameters (k). ĉ = 1.08 for the Pradel-models. Model notation: renest = nesting attempt (first nest vs. re-nest), age = age specific nest survival, t = year effect, (.) = no variation, hatch = hatching day (1st day was 27 of May), aci = i age classes with no other variation, aci[x]= x effect in age class i, TSM = time since marking, sex = sex of the individual, + = additive effect, * = interaction.

| No. | Daily nest survival | | AICc | | ΔAICc | w | | k | |  |
| --- | --- | --- | --- | --- | --- | --- | --- | --- | --- | --- |
| A1 | t*age | | 522.39 | | 0.00 | 0.671 | | 16 | |  |
| A2 | t*age+renest | | 523.81 | | 1.42 | 0.329 | | 17 | |  |
| A3 | t | | 539.31 | | 16.92 | 0.000 | | 9 | |  |
| A4 | t+age | | 539.32 | | 16.93 | 0.000 | | 10 | |  |
| A5 | t+age+renest | | 540.16 | | 17.77 | 0.000 | | 11 | |  |
| A6 | age+renest | | 552.99 | | 30.60 | 0.000 | | 3 | |  |
| A7 | age*renest | | 554.04 | | 31.65 | 0.000 | | 4 | |  |
| A8 | age | | 556.47 | | 34.08 | 0.000 | | 2 | |  |
| A9 | renest | | 556.94 | | 34.55 | 0.000 | | 2 | |  |
| A10 | . | | 561.32 | | 38.93 | 0.000 | | 1 | |  |
| No. | Φ Juvenile |  | AICc | | ΔAICc | w | | k | |  |
| B1 | Φ(ac1[c+hatch],ac2[c]) p(ac3) | | 430.215 | | 0 | 0.795 | | 6 | |  |
| B2 | Φ(ac2) p(ac3) | | 433.934 | | 3.72 | 0.124 | | 5 | |  |
| B3 | Φ(ac2) p(ac4) | | 435.778 | | 5.56 | 0.049 | | 6 | |  |
| B4 | Φ(ac1[t],ac2[c]) p(ac3) | | 438.913 | | 8.7 | 0.010 | | 12 | |  |
| B5 | Φ(ac1[t+hatch],ac2[c]) p(ac3) | | 439.048 | | 8.83 | 0.010 | | 13 | |  |
| B6 | Φ(ac2) p(ac2) | | 439.461 | | 9.25 | 0.008 | | 4 | |  |
| B7 | Φ(ac1[t],ac2[c]) p(ac4) | | 440.896 | | 10.68 | 0.004 | | 13 | |  |
| B8 | Φ(ac1[t],ac2[c]) p(ac2) | | 445.108 | | 14.89 | 0.000 | | 11 | |  |
| B9 | Φ(ac1[t*hatch],ac2[c]) p(ac3) | | 448.986 | | 18.77 | 0.000 | | 20 | |  |
| B10 | Φ(.) p(ac4) | | 461.474 | | 31.26 | 0.000 | | 5 | |  |
| B11 | Φ(.) p(ac3) | | 464.151 | | 33.94 | 0.000 | | 4 | |  |
| B12 | Φ(.) p(ac2) | | 501.158 | | 70.94 | 0.000 | | 3 | |  |
| B13 | Φ(ac2) p(.) | | 514.84 | | 84.62 | 0.000 | | 3 | |  |
| B14 | Φ(.) p(.) | | 654.349 | | 224.13 | 0.000 | | 2 | |  |
| No. | Φ Adult |  | AICc | | ΔAICc | w | | k | |  |
| C1 | Φ (.) p(.) | | 614.90 | | 0.00 | 0.236 | | 2 | |  |
| C2 | Φ (sex) p(.) | | 615.52 | | 0.62 | 0.172 | | 3 | |  |
| C3 | Φ (TSM) p(.) | | 616.14 | | 1.24 | 0.127 | | 3 | |  |
| C4 | Φ (.) p(sex) | | 616.26 | | 1.36 | 0.119 | | 3 | |  |
| C5 | Φ (sex+TSM) p(.) | | 616.85 | | 1.95 | 0.089 | | 4 | |  |
| C6 | Φ (sex) p(sex) | | 617.16 | | 2.26 | 0.076 | | 4 | |  |
| C7 | Φ (TSM) p(sex) | | 617.56 | | 2.66 | 0.062 | | 4 | |  |
| C8 | Φ (sex*TSM) p(c) | | 617.82 | | 2.92 | 0.055 | | 5 | |  |
| C9 | Φ (sex+TSM) p(sex) | | 618.53 | | 3.63 | 0.038 | | 5 | |  |
| C10 | Φ (sex*TSM) p(sex) | | 619.39 | | 4.49 | 0.025 | | 6 | |  |
| No. | λ / f |  |  | QAICc | ΔQAICc | | w | | k | |
| D1 | Φ (.) p(.) λ/f(t) | | 1291.73 | | 0 | 0.979 | | 10 | |  |
| D2 | Φ (t) p(.) λ/ f(t) | | 1300.78 | | 9.06 | 0.011 | | 17 | |  |
| D3 | Φ (.) p(t) λ/f(t) | | 1300.89 | | 9.17 | 0.010 | | 17 | |  |
| D4 | Φ (t) p(t) λ/f(t) | | 1309.33 | | 17.61 | 0.000 | | 23 | |  |
