# Supplementary material for: Grazed wet meadows are sink habitats for the southern dunlin (Calidris alpina schinzii) due to nest trampling by cattle
Source: Ecol Evol. 2016 Sep 9;6(20):7176–87. doi: 10.1002/ece3.2369 (PMC5513266; doi:10.1002/ece3.2369)
Supplement: Supplementary file 2 — Appendix S1. Methods on capture–recapture and nest survival models. [file ECE3-6-7176-s002.docx]

Supplementary Material, Appendix S1

Caption: Description of the methods regarding capture-recapture and nest survival modelling.

Pakanen, V.-M., Aikio, S., Luukkonen, A. & Koivula, K. 2016. Grazed wet meadows are sink habitats for the southern dunlin (*Calidris alpina schinzii*) due to nest trampling by cattle. *Ecology and Evolution*

Nest survival

We modelled daily nest survival (Dinsmore, White & Knopf 2002) in program MARK (White and Burnham 1999) considering all causes of nest failure excluding those caused by cattle see Pakanen, Luukkonen & Koivula (2011) for details. We assumed that nests damaged by cattle survived until the estimated time of failure. We included nests with unknown fate until the last known active date. Independent factors were year (2002 – 2010), nest age and nesting attempt (first or renest).

Apparent juvenile survival and age specific breeding probabilities

We analysed juvenile survival (from hatching to one year of age) from capture-recapture data collected from chicks ringed as hatchlings between 2002 and 2009. The data included 372 individuals of which 43 returned to the study area by 2010.The global model Φ(a1[t], a2[c]) p(4ac) included two age classes (hatching year [HY] and after hatching year [AHY]) for survival (Φ) and a time effect for juvenile survival (a1) but constant adult survival (a2), and four age classes (ac4) for recapture (p) probabilities. We included four age classes in the recapture probabilities for the purpose of estimating breeding probabilities (see below). Data were not sufficient to model temporal variation in addition to age effects on the recapture probabilities. The global model fit the data with no overdispersion (GOF_BOOTSTRAP_, p = 0.475, ĉ = 1.006). We examined the effect of hatching date on juvenile survival because possible temporal variation during the season is crucial for examining effects from trampling.

We estimated age (j) specific recruitment probability (B_j_) using age specific recapture probabilities pj (Clobert *et al*. 1994, Sanz-Aquilar *et al*. 2009). By assuming that full breeding (recruitment) is achieved by age k, the age specific recruitment probabilities can be calculated as pj/pk. This relies on the assumption that there is no reproductive skipping. There is no information suggesting that adults would not attempt to breed in some years.

Apparent adult survival

Adult data included 461 captures of 174 adult birds (49 % males). The global model including effects of time (t, year), time since marking (TSM, i.e. an age effect, two classes) and sex of the bird with full interactions (*), Φ(sex*TSM*t) p(sex*t) fit the data (Median-ĉ = 0.99, 95 % CI 0.82 – 1.16). We included a time since marking (i.e. an age effect) structure because first year survival after initial capture may be lower than after recaptures due to possible age or transience effects (Pradel *et al*. 1997).

References

Dinsmore, S.J., White, G.C. & Knopf, F.L. (2002) Advanced techniques for modeling avian nest survival. *Ecology,* **83**, 3476–3488.

Clobert, J., Lebreton, J.-D., Allaine, D. & Gaillard, J.M. (1994) The estimation of age-specific breeding probabilities from recaptures or resightings in vertebrate populations: II. Longitudinal models. *Biometrics,* **50**, 375–387.

Pakanen, V.-M., Luukkonen, A. & Koivula, K. (2011) Nest predation and trampling as management risks in grazed coastal meadows. *Biodiversity and Conservation,* **20**, 2057–2073.

Pradel, R., Hines, J.E., Lebreton, J.-D. & Nichols, J.D. (1997) Capture–recapture survival models taking account of transients. *Biometrics*, **53**, 88–99.

Sanz-Aguilar, A., Massa, B., Lo Valvo, F., Oro, D., Minguez , E. & Tavecchia, G. (2009) Contrasting age-specific recruitment and survival at different spatial scales: a case study with the European storm petrel. *Ecography,* **32**, 637–646.

White, G.C. & Burnham, K.P. (1999) Program MARK: survival estimation from populations of marked animals. *Bird Study*, **46**,120–139.
