# Supplementary material for: Grazed wet meadows are sink habitats for the southern dunlin (Calidris alpina schinzii) due to nest trampling by cattle
Source: Ecol Evol. 2016 Sep 9;6(20):7176–87. doi: 10.1002/ece3.2369 (PMC5513266; doi:10.1002/ece3.2369)
Supplement: Supplementary file 3 — Appendix S2. Description of the renesting model. [file ECE3-6-7176-s003.docx]

Supplementary Material, Appendix S2

Caption: Description of the renesting model that is used to calculate the number of successful females, the number of chicks that hatch and the local recruitment per female breeding attempt.

Pakanen, V.-M., Aikio, S., Luukkonen, A. & Koivula, K. (2016) Grazed wet meadows are sink habitats for the southern dunlin (*Calidris alpina schinzii*) due to nest trampling by cattle. *Ecology and Evolution*

The renesting model

We modeled the replacement of nests after a nesting failure when calculating the proportion of successful breeding attempts, number of hatchlings produced and local recruitment per female using a stochastic simulation model (renesting model, see Beintema & Müskens 1987). For females (replicates), the model of the whole breeding cycle started from egg laying and considered different parameters affecting breeding in sequence until the recruitment of juveniles for allowing parameters to be functions of date of the season. The replicates were then used to calculate averages across the population. The model was parameterized with life history data acquired and reported in this study. The final product, local recruitment (LR), derived with the renesting model describes the number of one year old birds produced by a female per breeding attempt:

Eq. 1. LR = NS * HS * Sjuv + (1 – NS) * RP * NS *HS * Sjuv,

where NS = nest success, HS = hatching success, Sjuv = survival from hatching to age one and RP = renesting probability. The parameters NS, Sjuv and RP were temporally variable over the breeding season.

In the first stage of the model, each female was assigned a date of nest initiation by randomly drawing from the observed distribution (**a**, Fig. S1; Supplementary material, Appendix S4, Fig S3). After nest initiation, daily fates of the nests until hatching (26 days) were determined by nest age specific survival (Supplementary material, Appendix S4, Fig S5). Nest fate was determined at each simulated day by comparing the daily nest survival probability to a random number drawn from a uniform distribution (**b**, Fig. S1). The daily nest survival probability was drawn from a Beta distribution using the Alfa and Beta parameters that were calculated with the daily mean and variance estimates. If the random probability was higher than the drawn nest survival probability, the nest became destroyed. These nest survival estimates do not include the effect of trampling, which is modelled separately. The nesting period (**P**, Fig. S1), including both laying and incubation stages, lasted 26 days (Soikkeli 1967). If the nest was destroyed during this period, the female was assigned to lay a replacement with a probability that decreased with the advancement of the breeding season (**c**, Fig. S1; Supplementary material, Appendix S4, Fig. S4).

The observed mean re-laying interval between nest failure and the first egg of the replacement clutch was used as the expected value of the Poisson-distributed re-laying interval in the simulation that determined the date of nest replacement (**d**, Fig. S1). The maximum number of possible nesting attempts per individual was set at two. The last day of nest initiation was set to be 15^th^ of June. The number of hatchlings per nest in the simulation was defined as the observed frequency of chick numbers [fq(n)] (**e**, Fig. S1). Recruitment to the population was the product of chick number and calendar day specific juvenile survival (**f**, Fig. S1; Supplementary material, Appendix S4, Fig S6).

In scenarios including trampling, nest fate from trampling was determined after that of losses to other causes (**g**, Fig. S1). Trampling is most severe at the start of grazing, when the stock moves most actively on the pasture, and declines gradually during the first three weeks of grazing (Pakanen, Luukkonen & Koivula 2011). This temporal variation was modeled with a time dependent function based on the slope estimate described in Pakanen, Luukkonen & Koivula (2011, Fig. S2). Trampling rates were assumed to stabilize after the second week. All scenarios were examined for each potential grazing initiation date beginning from 1st of May ending until 30th of June. We present averages of 10000 replicated simulations for each initiation date.

Fig. S1 Flow chart of the renesting model that is used to calculate the proportion of successful breeding attempts, number of hatchlings produced and local recruitment per female southern dunlin

START

(**a**) Set laying date (i)

P = P-1

i = i+1

Is P > 0?

yes

yes

Nest hatched

END

Add to hatched clutches

Breeding attempt failed

(**c**)

Fig. in Pakanen et al., (2011;Clutch replaced?

(**d**)

Set day of replacement

yes

no

no

no

(**b**)

Does nest survive day *i* from predation

yes

(**g**)

Does nest survive day i from trampling

no

Are there

more females?

yes

Next female

no

Nesting period (**P**) begins

(**e**) Set clutch size

(**f**) Calculate number of local recruits

Add to clutch sizes

Add to number of local recruits


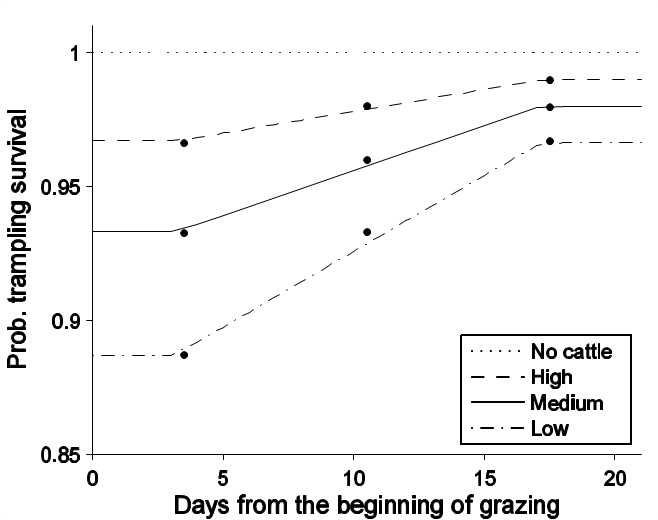


Fig. S2 Survival rates from trampling used in different scenarios (no cattle, high survival = low stocking rates, medium survival = moderate stocking rate and low survival = high stocking rates) in the renesting model in relation to time since start of grazing. The data for low survival (high trampling; 1.72 head/ha) are derived from Pakanen, Luukkonen & Koivula (2011). The medium (0.96/day) and high (0.98 per day) survival probabilities (medium [1.0 head/ha] and low [0.5 head/ha] trampling rates, respectively) were drawn from literature (Beintema & Müskens 1987; Thorup 1998; Pakanen, Luukkonen & Koivula 2011) and modeled with a time dependent function based on the slope estimate described in Pakanen, Luukkonen & Koivula (2011). The dots represent these estimates, and the curves represent linear regressions over their ranges. Trampling survivals are extrapolated as constants outside the range of observed values

References

Beintema, A.J., Müskens, G.J.D.M. (1987) Nesting suc­cess of birds breeding in Dutch agricultural grasslands. *Journal of Applied Ecology*, **24,** 743–758.

Pakanen, V.-M., Luukkonen, A. & Koivula, K. (2011) Nest predation and trampling as management risks in grazed coastal meadows. *Biodiversity and Conservation,* **20**, 2057–2073.

Soikkeli, M. (1967) Breeding cycle and population dynamics in the dunlin (Calidris alpina). *Ann. Zool. Fenn*. **4**, 158–198.

Thorup, O. (1998) Ynglefuglene på Tipperne 1928-1992. *Dansk Ornitologisk Forenings Tidsskrift,* **92**, 1–192.
