# Supplementary material for: Grazed wet meadows are sink habitats for the southern dunlin (Calidris alpina schinzii) due to nest trampling by cattle
Source: Ecol Evol. 2016 Sep 9;6(20):7176–87. doi: 10.1002/ece3.2369 (PMC5513266; doi:10.1002/ece3.2369)
Supplement: Supplementary file 4 — Appendix S3. Description of the matrix model and the PVA. [file ECE3-6-7176-s004.docx]

Supplementary Material, Appendix S3

Caption: Description of the matrix model and the population viability analysis.

Pakanen, V.-M., Aikio, S., Luukkonen, A. & Koivula, K. (2016) Grazed wet meadows are sink habitats for the southern dunlin (*Calidris alpina schinzii*) due to nest trampling by cattle. *Ecology and Evolution*

We estimated the population growth rate as the dominant eigenvalue of a Lefkovich matrix model (Caswell 2001) that was based on a pre-breeding census and female dynamics with stages representing 3 age groups (1, 2 and 3 years old or older). The matrix and its multiplication are written as

Eq. 1. $\left[\begin{matrix} N_{1} \\ N_{2} \\ N_{3} \end{matrix}\right]_{t+1}=\left[\begin{matrix} F_{1} & F_{2} & F_{3} \\ S & 0 & 0 \\ 0 & S & S \end{matrix}\right]\left[\begin{matrix} N_{1} \\ N_{2} \\ N_{3} \end{matrix}\right]_{t}$,

${\text{Eq. 1 }\left[ \begin{matrix} \text{N}_{1} \\ \text{N}_{2} \\ \text{N}_{3} \\ \text{N}_{4} \end{matrix} \right]}_{\text{t+1}}\text{= }\left[ \begin{matrix} 0 & F_{2} \\ 1 & 0 \\ 0 & \text{S} \\ 0 & 0 \end{matrix}\text{ }\begin{matrix} F_{3} & F_{4} \\ 0 & 0 \\ 0 & 0 \\ \text{S} & \text{S} \end{matrix} \right]\text{ }\left[ \begin{matrix} \text{N}_{1} \\ \text{N}_{2} \\ \text{N}_{3} \\ \text{N}_{4} \end{matrix} \right]_{\text{t}}\text{+ }\left[ \begin{matrix} 0 \\ 0 \\ 0 \\ \text{I} \end{matrix} \right],$where F_i_ express age dependent fecundities, i.e. the mean number of recruits produced per female and S is the survival from one year to the next did not change with age. Fecundities were calculated as

Eq. 2 F_i_ = ½ * BPi * LR,

where BP is breeding probability at age i (age specific recruitment), and LR is local recruitment per breeding female calculated with the renesting model (Supplementary material, Appendix S2). LR describes the number of one year old birds produced by a female per breeding attempt:

Eq. 3. LR = NS * HS * Sjuv + (1 – NS) * RP * NS *HS * Sjuv,

where NS = nest success, HS = hatching success, Sjuv = survival from hatching to age one and RP = renesting probability. Sex ratios were set even (own unpublished data). We calculated the variance for lambda as,

Eq. 4 $Var(\lambda)= \sum\left(\frac{\partial\lambda}{\partial\theta}\right)^{2}\sigma_{\theta}^{2}$

where $\sigma_{\theta}^{2}$is the variance of a matrix element and $\frac{\partial\lambda}{\partial\theta}$ is the sensitivity of lambda to this element (Lande 1988). The variances were calculated from annual element values.

The matrix model did not include the effects of immigration. We considered the impact of immigration to population growth analytically by calculating equilibrium population size **Ň** = (**I–A**^-1^)**n**, where **A** is the transition matrix and **I** is an identity matrix of corresponding size. Vector **n** indicates the number of immigrants to each stage of the population. We parameterized immigration with the mean number immigrants, and directed it to the oldest age group in which breeding probability was 100%, i.e., **n** = [0 0 I]^T^ (Eq. 1). Matrix models were constructed in program MATLAB (The MathWorks Inc.). Immigration rate derived from the results of this study.

Examination of extinction risk

We used RAMASmetapop (Akçakaya 2005) to analyze population viability under variable grazing scenarios using the matrix model described above. Density dependence affects survival of dunlin (Ryan et al. 2016), but we could not include it in the model due to lack of data to parameterize it with sufficient accuracy. Density dependence of long distance migratory waders is a problematic parameter because the group of individuals affecting fitness differs from the breeding population we are examining. Density dependence is strongest at the non-breeding sites, where all species compete for resources and most mortality occurs (Leyrer et al. 2013, Ryan et al. 2016). Thus, it should be safe to use the ceiling type of density dependence, where we set the carrying capacity at 200 individuals. We assumed that fecundities and survival rates correlate because juvenile survival and adult survival are affected by the same conditions during the non-breeding season. Uncertainty about future vital rates and the nature of density dependence affecting population dynamics at small (e.g. Allee effects) or large population sizes limits our ability to make long term predictions. We therefore projected population growth only for the near future, i.e. next 20 years, which is about three to four dunlin generations (White 2000). We used a threshold of 30 % decline to examine population viability when immigration was included in the model. When immigration was not included, we used an extinction threshold of one individual. Because variation from all environmental factors affecting vital rates (e.g. flooding) should be included in the parameter estimates, we did not model catastrophes. Initial population size was set to 47 pairs observed in 2010 and a stable stage distribution was assumed.

References

Akçakaya, H.R. (2005) *RAMAS GIS: Linking spatial data with population viability analysis (version 5)*. Setauket, New York. Applied Biomathematics.

Caswell, H. (2001) *Matrix Population Models*. Sinauer.

Leyrer, J., Lok, T., Brugge, M., Spaans, B., Sandercock, B.K. & Piersma, T. (2013) Mortality within the annual cycle: seasonal survival patterns in Afro-Siberian red knots *Calidris canutus canutus*. *J Ornithol* **154**,933-943

Ryan, L.J., Green, J.A. & Dodd, S.G. (2016) Weather conditions and conspecific density influence survival of overwintering Dunlin *Calidris alpina* in North Wales. *Bird Study*, **63**, 1–9.

White, G.C. (2000) Population viability analysis: data requirements and essential analyses. In: Boitani L, Fuller TK (eds) Research techniques in animal ecology: controversies and consequences, Columbia University Press, New York, USA, pp 288–331
