# Supplementary material for: Grazed wet meadows are sink habitats for the southern dunlin (Calidris alpina schinzii) due to nest trampling by cattle
Source: Ecol Evol. 2016 Sep 9;6(20):7176–87. doi: 10.1002/ece3.2369 (PMC5513266; doi:10.1002/ece3.2369)
Supplement: Supplementary file 5 — Appendix S4. Results as figures. [file ECE3-6-7176-s005.docx]

Supplementary Material, Appendix S4

Caption: Result figures of timing of breeding, nest survival, renesting probability, juvenile survival and evaluating a sink population status.

Pakanen, V.-M., Aikio, S., Luukkonen, A. & Koivula, K. (2016) Grazed wet meadows are sink habitats for the southern dunlin (*Calidris alpina schinzii*) due to nest trampling by cattle. *Ecology and Evolution*

Fig. S3. Date of laying first egg for first nests (white bars, n = 254) and renests (black bars, n = 26) of Southern dunlin breeding at Bothnian Bay during 2002 to 2010.

Fig. S4. Renesting probability in relation to date of nest failure (logistic regression, z = -3.647, p < 0.0002), observed (filled circles) and fitted logistic regression curve (log(y/(1–y)) = 2.52364 + date*0.11869) that was used in the simulation model for the temporal change in renesting probability.

Fig. S5. Mean daily nest survival (±SE) of Southern dunlin for different years (estimates derived from model A3 in Table S1).

Fig. S6. Daily nest survival (±SE) of Southern dunlin nests as a function of nest age for different years.

 Fig. S7. Apparent juvenile survival of Southern dunlin chicks (±SE) from 2002 to 2008. Note that these estimates were drawn from model B4, not by model averaging.

Fig. S8. Apparent juvenile survival of Southern dunlin chicks (±SE) as a function of calendar hatching date.

Fig. S9. Apparent adult survival of Southern dunlin (±SE) males (open circles) and females (black squares) from 2002 to 2010. Note that these estimates were drawn from model Φ(t+sex)p(c). The estimates do not take into account time since marking and were not estimated by model averaging.


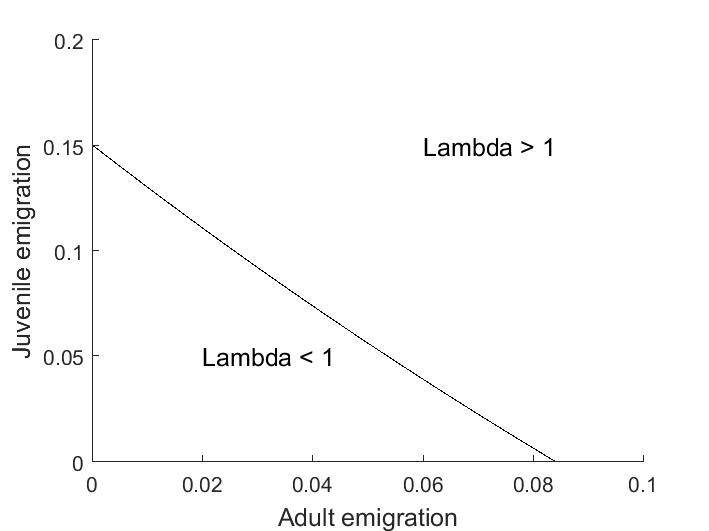


Fig. S10 Classification of the study population to a source or a sink under different combinations of juvenile and adult emigration rates. A stable population (λ = 1) is indicated by the line below which the population is considered a sink and when above as a source, Emigration is comparable with survival, i.e. a 0.1 emigration rate is equal to the same “mortality rate” in terms of population growth.
